# Transcranial Direct Current Stimulation for Treatment of Childhood Pharmacoresistant Lennox–Gastaut Syndrome: A Pilot Study

**DOI:** 10.3389/fneur.2016.00066

**Published:** 2016-05-04

**Authors:** Narong Auvichayapat, Katenipa Sinsupan, Orathai Tunkamnerdthai, Paradee Auvichayapat

**Affiliations:** ^1^Department of Pediatrics, Division of Pediatric Neurology, Faculty of Medicine, Khon Kaen University, Khon Kaen, Thailand; ^2^Department of Physiology, Division of Neuroscience, Faculty of Medicine, Khon Kaen University, Khon Kaen, Thailand

**Keywords:** transcranial direct current stimulation, cathodal stimulation, childhood pharmacoresistant epilepsy, Lennox–Gastaut syndrome

## Abstract

**Background:**

Lennox–Gastaut syndrome (LGS) is a severe childhood epileptic syndrome with high pharmacoresistance. The treatment outcomes are still unsatisfied. Our previous study of cathodal transcranial direct current stimulation (tDCS) in children with focal epilepsy showed significant reduction in epileptiform discharges. We hypothesized that cathodal tDCS when applied over the primary motor cortex (M1) combined with pharmacologic treatment will be more effective for reducing seizure frequency in patients with LGS than pharmacologic treatment alone.

**Materials and methods:**

Study participants were randomized to receive either (1) pharmacologic treatment with five consecutive days of 2 mA cathodal tDCS over M1 for 20 min or (2) pharmacologic treatment plus sham tDCS. Measures of seizure frequency and epileptic discharges were performed before treatment and again immediately post-treatment and 1-, 2-, 3-, and 4-week follow-up.

**Result:**

Twenty-two patients with LGS were enrolled. Participants assigned to the active tDCS condition reported significantly more pre- to post-treatment reductions in seizure frequency and epileptic discharges that were sustained for 3 weeks after treatment.

**Conclusion:**

Five consecutive days of cathodal tDCS over M1 combined with pharmacologic treatment appears to reduce seizure frequency and epileptic discharges. Further studies of the potential mechanisms of tDCS in the LGS are warranted.

**Trial Registration:**

ClinicalTrials.gov, NCT02731300 (https://register.clinicaltrials.gov).

## Introduction

Lennox–Gastaut syndrome (LGS) is a severe childhood epileptic syndrome that is characterized by multiple types of seizures including a nucleus of brief tonic, atonic seizures, atypical absences, and less characteristically, myoclonic attacks associated with an interictal electroencephalography (EEG) pattern of diffuse, slow spike–wave complexes <2.5 Hz (1). The prevalence of LGS was approximately 3–10.7% of all cases of childhood epilepsies (2, 3), and the incidence reported in Finland was 2/100,000 among children aged 1–14 years (4). Almost all LGS are mentally retarded (5, 6). This syndrome is highly pharmacoresistant (7) and required non-pharmacologic interventions such as ketogenic diet, vagus nerve stimulation, and epilepsy surgery including callosotomy and focal curative resection that takes risk and ineffective (8).

Transcranial direct current stimulation (tDCS) is a painless and safe method for focal brain stimulation (9). tDCS is based on decade-old observations that neuronal firing can be modulated by low amplitude electrical direct current (10). Even though the electrical current used in tDCS is low, it is large enough to decrease the threshold needed to generate hyperpolarization in neurons immediately below the cathodal (negative) electrode. Thus, it is thought to modulate cortical excitability by altering cell membrane potential. Although the precise mechanisms that underlie tDCS are not yet completely understood, the overall effect on the human cortex is reliable, such that anodal tDCS facilitates cortical activity and cathodal tDCS depresses cortical activity (11).

Transcranial direct current stimulation units are light weight and inexpensive. It is presently under investigation for epilepsy treatment, where excess cortical excitability is a prominent feature and neuronal inhibition from cathodal tDCS may be beneficial. For treatment of seizures, tDCS may offer a practical therapeutic option with the benefit of easy, rapid, and focal application in the setting of acute seizure or other form of brain injury (12, 13).

A previous study has suggested that cortical excitability observed in LGS was decreased when compared to patients with chronic refractory focal epilepsy and healthy non-epilepsy controls (14). Recently, a number of studies have suggested that cathodal tDCS may be useful in suppressing seizures. A previous study of tDCS in a focal epilepsy rodent model showed a significant elevation of the seizure threshold (15) and decreased convulsions following pilocarpine-induced status epilepticus in rats (16). A clinical trial of cathodal tDCS in 19 adult patients with refractory epilepsy showed the effective suppression of epileptiform discharges on EEG, but only a trend toward clinical improvement (17). In addition, our previous study of a single cathodal tDCS in 36 children with refractory focal epilepsy also showed significant reductions in epileptic discharge immediately and both 24 and 48 h after tDCS. The seizure frequency was also decreased for 4 weeks after treatment (18).

As mentioned previously, no research has yet examined the effects of cathodal tDCS for LGS. However, given that LGS may share some pathophysiology with refractory focal epilepsy that could be response to anti-epileptic effect, specifically, the action on, epileptic neurons and synchronization (19, 20). It is reasonable to examine the potential effects of cathodal tDCS on LGS. We hypothesized that five consecutive days of cathodal tDCS would result in significantly more pre- to post-treatment decreases in seizure frequency than 5 days of sham tDCS stimulation, and that the differences in seizure frequency would maintain for at least 4 weeks post-treatment. Therefore, the objective of our study was to test the efficacy of tDCS in the pediatric LGS population.

## Materials and Methods

### Participant Recruitment and Informed Consent

Study participants were recruited *via* advertisement at the pediatric outpatient department, Srinagarind hospital, Faculty of Medicine, Khon Kaen University, Thailand. The study procedures were described to any eligible patients who expressed an interest in participating in the study by a pediatric neurologist. Criteria for LGS were defined according to the triad of (1) polymorphic intractable seizures that are mainly tonic, atonic, and atypical absence seizures, (2) cognitive and behavioral abnormalities, (3) EEG with paroxysms of fast activity and slow (<2.5 Hz) generalized spike-wave discharges (GSWD) (21). The diagnosis was confirmed by a pediatric neurologist using the thoroughly history taking, physical examination, EEG, and brain MRI. Study inclusion criteria included (a) diagnosis of LGS; (b) failure of more than two first-line AEDs to control seizures; (c) average seizure frequency of more than one per month for 18 months and no more than three consecutive seizure-free months during that interval; (d) age between 6 and 15 years. The exclusion criteria were (a) drug addiction, pregnancy, skull defect, and other serious neurological diseases; and (b) change in dosage of antiepileptic drugs or use of herbal remedies and other alternative therapies.

All patients’ guardians gave their written informed consent. The study conformed to the declaration of Helsinki and was approved by the Ethics Committee of Khon Kaen University (Identifier number: HE 521232).

### Experimental Design

The current study was a randomized, double-blind controlled trial performed over a total of 6 weeks consisting of (1) a 1-week period of observation to assess the baseline seizure frequency, (2) a five consecutive days of 2 mA cathodal tDCS for 30 min, and (3) 4 weeks of follow-up. Just before the treatment phase, study participants were randomized in a 2:1 ratio in blocks of four randomizations to receive either (a) pharmacologic treatment plus active tDCS stimulation or (b) pharmacologic treatment plus sham tDCS stimulation for 5 days. Participants were asked to continue their routine anti-epileptic medication regimen throughout the duration of the 6-week trial.

### Pharmacologic Treatment

Since there is a considerable degree of heterogeneity with LGS, individualized approaches are necessary. We first use the antiepileptic drugs that currently approved by the Food and Drug Administration and available in our institute: lamotrigine, topiramate, clobazam, and clonazepam depended on seizure types. We gave lamotrigine 1–20 mg/kg/day with slowly titration for tonic, tonic–clonic, atypical absences, and atonic seizures. Clobazam 0.25–3.5 mg/kg/day was added in the cases that refractory to lamotrigine and in the patients with myoclonic seizures. We used clonazepam 0.04–0.2 mg/kg/day instead of clobazam in some cases when clobazam was not available. We used topiramate 1–10 mg/kg/day in the patients who refractory to lamotrigine and clobazam. In some cases, that refractory to the aforementioned antiepileptic drugs, we gave zonisamide 1–20 mg/kg/day, levetiracetam 10–80 mg/kg/day, and nitrazepam 0.1–0.8 mg/kg/day (8).

#### Randomization and Blinding

Prior to the treatment phase, study participants were randomized in a 2:1 ratio in blocks of four randomizations (by OT) to receive either (1) active tDCS stimulation or (2) sham tDCS stimulation. Participants were asked to continue their routine medication regimen throughout the trial. The staff who generated the random allocation sequence, enrolled participants, and assigned participants to interventions were not involved in any assessments. After assignment to the intervention groups, the pediatric neurologist who carried out the seizure assessments (Narong Auvichayapat) was blind to treatment condition. Because the study participants were also blind to treatment condition, this is a double-blind study.

#### Active and Sham Transcranial Direct Current Stimulation

Transcranial direct current stimulation was applied *via* water-soaked pair of surface sponge electrodes (35 cm^2^) and delivered through battery-driven power supply. The constant current stimulator had a maximum output of 10 mA (Soterixmedical, Model 1224-B, New York, NY, USA). The stimulation site over the left M1, located based on the international electroencephalography (EEG) 10/20 electrode placement system. The reference electrode was placed over the right shoulder area.

The tDCS device was designed to allow sham stimulation by placing the control switch in front of the instrument, which was easily covered by an opaque adhesive during stimulation. Therefore, the patients or their gradients could not know whether active or sham stimulation. The power lit up indicator was also on the front of the machine during the time of intervention both in active and sham stimulations. However, in sham stimulation, the current was discontinued after 30 s, while the power indicator remained on (18).

### Measures

#### Number of Seizures

Since Lennox–Gastaut Syndrome composed of many seizure types. All seizures were classified according to the International League Against Epilepsy Revised Classification of Seizures (22). The patients were monitored by video-EEG in order to classify the types and frequencies of seizures, and all of the caregivers were taught how to count and classify seizures on the basis of observations and video recordings. The caregivers were also taught the rules for diary recording prior to the baseline period. All of the caregivers were blinded to both treatment and sham group. In case of the patient who had more than one caregiver, we suggested them to refer the daily diary to the person who cared the patient in time.

Number of seizures was the primary outcome variable, and was assessed by using a daily diary. For the baseline (pre-treatment) assessment, the caregivers were asked to record the number of seizures every day for 7 days during the baseline period on a daily diary. These 7 days, numbers of seizures were averaged into a single rate per day of baseline average seizure frequency. During the five consecutive days of treatment, the caregivers were asked to record the daily children seizure frequency. Finally, daily 24-h recording were administered for 4 weeks following treatment. 1-, 2-, 3-, and 4-week composite number of seizures were computed as an average of the daily number of seizures for each epoch (i.e., the 1-week follow-up average number of seizures = average of seven daily number of seizures the first week after treatment).

#### Epileptic Discharges

Epileptic discharges, the secondary outcome variable of this study, were recorded by a trained staff. EEG was acquired from all patients using a 32-channel, international 10–20 system of electrode placement (Neuvo, Compumedics, Australia with PerFusion EEG software). EEG was collected for 30 min in the awake state only. EEG was recorded as a single session at baseline, immediately, 1-, 2-, 3-, and 4-week follow-up. EEG data were analyzed by visual inspection. The numbers of epileptic discharges in the 30-min recording were assessed by a practicing pediatric neurologist and clinical neurophysiologist (Narong Auvichayapat), who was blinded to the treatment condition. The EEGs that included as abnormal EEGs in LGS are slow spike–wave complexes at <3 Hz that occur during wakefulness. The complexes consist of a spike (duration <70 ms) or a sharp wave (70–200 ms), followed first by a positive deep trough and then a negative wave (350–400 ms). Paroxysmal fast rhythms (10–20 Hz) occur mainly during non-rapid eye movement sleep (23). One slow spike–wave complex or one episode of paroxysmal fast rhythms were counted as one epileptic discharge.

#### Vital Signs and Oxygen Saturation Monitoring

All patients were closely observed by physicians during and post-treatment. Oxygen saturation and vital signs were monitored for 30 min before, during, and 30 min after the treatment. Pulse rate was measured by automatic sphygmomanometer (Ua-767 Plus, UK) in the supine position. Blood pressure (millimeter of mercury) was measured by automatic sphygmomanometer (Ua-767 Plus, UK) in the supine position with a pediatric-size cuff wrapped around the right upper arm. Body temperature was measured by an axillary electronic thermometer. Respiratory rate was measured by counting chest risings for 60 s. Pulse oximeter was placed on the left index finger to monitor oxygen saturation throughout the procedure.

#### Adverse Events

Adverse events as well as other signs and symptoms were reported by patients’ guardians every day after treatment. Theses self-recordings terminated at 4 weeks after stimulation.

### Statistical Analysis

We first computed means and SDs of the demographic and outcome variables for descriptive purposes. Next, we compared the two treatment conditions (active tDCS vs. sham tDCS) on all baseline outcome measures to ensure baseline equivalence using *t*-tests. Results are presented as means and SD. Both the primary (seizure frequency) and exploratory (epileptic discharges) hypotheses were tested using repeated measures analysis of variance (ANOVA) followed by LSD to help understanding any significant effects found. To describe the clinical meaningfulness of any changes, we computed the percent reduction of number of seizures and epileptic discharges in each condition from pre- to post-treatment and from pre-treatment to 4-week follow-up. For all analyses, *p* values of <0.05 were considered statistically significant. Analyses were completed using STATA software, version 10.0 (StataCorp, College Station, TX, USA).

## Result

**Figure 1:**
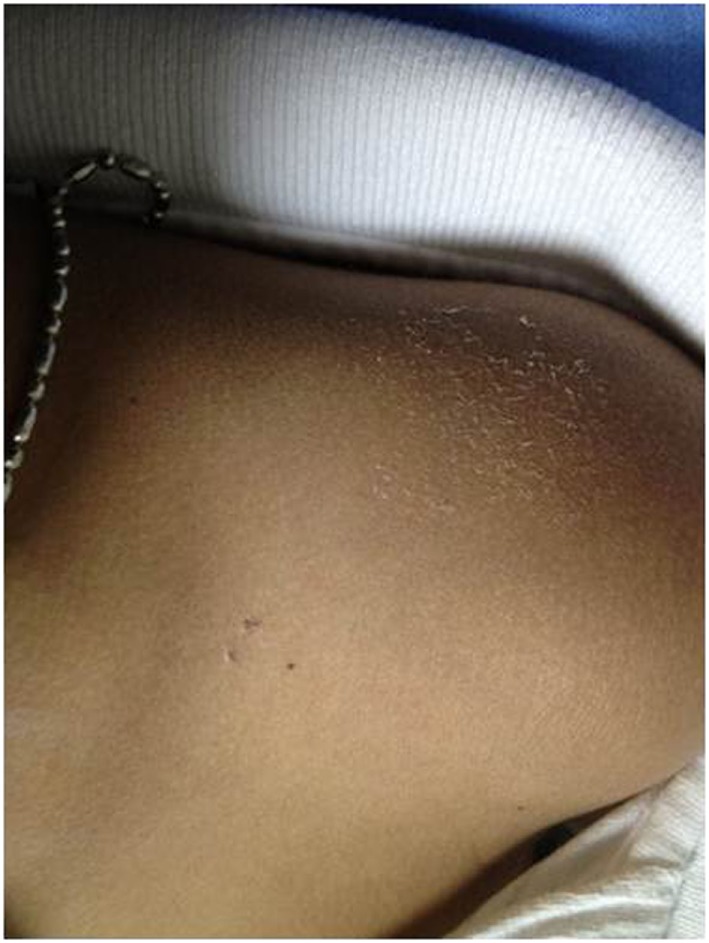
**Image of a single adverse event**. Three points of superficial skin burn under the reference electrode in active tDCS group, which resolved within 2 days.

The demographic data and baseline patient characteristics are described in Table 1. A total of 22 patients with LGS were enrolled between August 2010 and December 2013 (Table 1). All patients completed the entire protocol and tolerated the tDCS well, without serious adverse events. Only one patient had three points of 1 mm superficial skin burn under the reference electrode at the day 5 of the treatment period (Figure 1). However, the lesion spontaneously resolved in 2 days without scar lesion, no other serious side effect was observed.

**Table 1:** **Demographic data and baseline characteristics (*n* = 22)**.

	Cathodal tDCS	Sham
No. of subjects	15	7
Sex (males/females)	9/6	5/2
Age		
Mean ± SD	6.67 ± 1.54	6.29 ± 1.98
Range (years)	4–9	3–9
Etiologies of epilepsies		
Idiopathic	6	2
Infantile spasms	1	1
Neonatal hypoglycemia	4	3
Preterm with severe birth asphyxia	2	0
Preterm with history of intracerebral hemorrhage	2	1
Baseline seizure frequency per day (mean ± SD)	80.67 ± 54.43	93.43 ± 59.94
Baseline epileptic discharges per 30 min (mean ± SD)	640.13 ± 263.30	800.86 ± 374.62
Age at onset of seizures (years)	2.32 ± 2.39	1.58 ± 1.70
Number of antiepileptic drugs used		
3	9	3
4	4	2
5	2	2

### Clinical Seizure Reduction after tDCS

The repeated-measures ANOVA using the number of seizures as the dependent variable revealed significant main effects for group [*F* (1, 20) = 63.384; *p* < 0.001], time [*F* (9, 20) = 9.813; *p* < 0.001], and group × time interaction [*F* (9, 20) = 10.023; *p* < 0.001].

**Figure 2:**
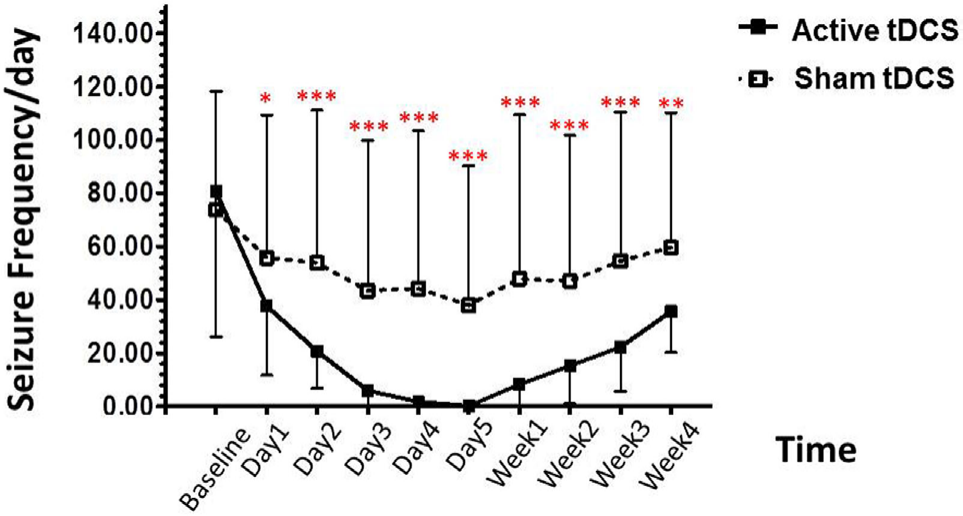
**Effect of tDCS on seizure frequency**. Data are presented as mean ± SD of number seizure frequency/day at baseline, treatment period (day 1–day 5), and various time points after treatment: 1, 2, 3, and 4 weeks. *p = 0.004, **p = 0.002, and ***p < 0.001 as compared to sham tDCS condition.

*Post hoc* analysis showed that the seizure frequency was significantly decrease in the tDCS group, relative to the sham tDCS group during the treatment period, at day 1 (*p* = 0.004), day 2 (*p* < 0.001), day 3 (*p* < 0.001), day 4 (*p* < 0.001), day 5 (*p* < 0.001). In addition, we also found significant decrease in seizure frequency at immediately (*p* < 0.001), 1 week (*p* < 0.001), 2 week (*p* < 0.001), 3 week (*p* < 0.001), and 4 week (*p* = 0.002) (Figure 2).

In the active tDCS group, the baseline for seizure frequency was 80.67 ± 54.43/day. At the treatment period; decreased to 37.60 ± 25.96/day (53.39% reduction), 20.60 ± 13.71/day (74.46% reduction), 5.87 ± 7.31/day (92.72% reduction), 1.73 ± 4.18/day (97.85% reduction), and 0.133 ± 0.52/day (99.84% reduction) after the 1, 2, 3, 4, and 5 days of treatment.

After the treatment, the seizure frequency was 8.27 ± 24.23/day (89.75% reduction), 15.20 ± 14.26/day (81.16% reduction), 22.27 ± 16.51/day (72.39% reduction), and 35.53 ± 15.29/day (55.96% reduction) in 1-, 2-, 3-, and 4-week follow-up, respectively.

### The Numbers of Individual Seizure Types

The mean numbers of baseline tonic, atonic, absence, myoclonic, and partial seizures in both groups are 22.68, 23.63, 21.32, 13.36, and 3.27, respectively. Cathodal tDCS reduced the seizure frequency by more than 50% in all individual seizure types. However, the between group analysis reveal statistically significant decrease in mean seizure frequency in only tonic, atonic, and absence seizures (Table 2).

**Table 2:** **The numbers of individual seizure types and percentage of seizure reduction**.

Seizure type	Active tDCS			Sham tDCS			*p* Value
	*n*	Mean (SD)	% Seizure reduction	*n*	Mean (SD)	% Seizure reduction	
**Tonic seizures**							
Baseline frequency	9	21.27 (21.67)		5	25.71 (22.63)		0.663
Day 1	8	9.40 (12.73)	55.81	5	24.00 (23.61)	6.65	0.071
Day 2	7	5.00 (7.11)	76.49	5	26.57 (22.98)	−3.35	0.003**
Day 3	2	0.47 (1.25)	97.79	5	25.29 (21.08)	1.63	0.000***
Day 4	2	0.27 (0.80)	98.73	5	28.57 (23.13)	−11.12	0.000***
Day 5	1	0.20 (0.77)	99.06	5	24.86 (21.48)	3.31	0.000***
Week 1	3	0.33 (0.72)	98.45	5	29.57 (24.75)	−15.01	0.000***
Week 2	7	2.00 (2.80)	90.60	5	25.43 (22.52)	1.09	0.001**
Week 3	7	5.07 (8.13)	76.16	5	29.57 (24.75)	−15.01	0.001**
Week 4	8	9.33 (12.26)	56.14	5	26.29 (22.88)	−2.26	0.033*
**Atonic seizures**							
Baseline frequency	7	19.20 (31.76)		5	33.14 (36.32)		0.370
Day 1	6	8.00 (13.49)	58.33	5	32.71 (33.79)	1.30	0.022*
Day 2	6	3.80 (6.11)	80.21	5	36.57 (38.32)	−10.35	0.003**
Day 3	5	2.67 (3.45)	86.09	5	32.14 (33.18)	3.02	0.002**
Day 4	1	0.27 (1.03)	98.59	5	34.57 (33.07)	−4.32	0.001**
Day 5	0	0.00 (0.00)	100.00	5	26.57 (24.24)	19.82	0.000***
Week 1	3	3.60 (12.08)	81.25	5	37.29 (37.84)	−12.52	0.005**
Week 2	7	4.00 (7.60)	79.17	5	32.14 (33.53)	3.02	0.005**
Week 3	7	4.80 (8.25)	75.00	5	38.00 (41.03)	−14.67	0.006**
Week 4	7	7.40 (9.55)	61.46	5	33.86 (38.50)	−2.17	0.019*
**Absence seizures**							
Baseline frequency	9	20.47 (22.97)		4	23.14 (26.56)		0.811
Day 1	9	9.60 (12.02)	53.10	4	25.57 (28.73)	−10.50	0.076
Day 2	9	5.80 (6.55)	71.67	4	25.71 (29.09)	−11.11	0.018*
Day 3	4	1.33 (2.50)	93.50	4	23.00 (26.43)	0.61	0.004**
Day 4	4	0.80 (1.52)	96.09	4	23.57 (26.16)	−1.86	0.002**
Day 5	2	0.53 (1.60)	97.41	4	23.86 (26.22)	−3.11	0.002**
Week 1	2	1.80 (5.95)	91.21	4	21.86 (25.78)	5.53	0.008**
Week 2	7	4.27 (6.26)	79.14	4	23.86 (27.82)	−3.11	0.015*
Week 3	8	5.53 (7.24)	72.98	4	23.14 (27.12)	0.00	0.026*
Week 4	8	8.60 (10.52)	57.99	4	21.00 (24.61)	9.25	0.108
**Myoclonic seizures**							
Baseline frequency	7	15.93 (21.31)		1	7.86 (20.79)		0.414
Day 1	7	8.67 (12.97)	45.57	1	8.57 (22.68)	−9.03	0.990
Day 2	7	5.67 (7.84)	64.41	1	7.14 (18.90)	9.16	0.795
Day 3	5	2.13 (3.74)	86.63	1	8.00 (21.17)	−1.78	0.299
Day 4	2	0.80 (2.24)	94.98	1	9.00 (23.81)	−14.50	0.189
Day 5	0	0.00 (0.00)	100.00	1	7.71 (20.41)	1.91	0.147
Week 1	3	2.53 (6.94)	84.12	1	8.86 (23.43)	−12.72	0.338
Week 2	6	4.20 (6.66)	73.63	1	7.86 (20.79)	0.00	0.536
Week 3	6	5.93 (8.71)	62.77	1	7.00 (18.52)	10.94	0.854
Week 4	6	8.20 (12.07)	48.52	1	8.00 (21.17)	−1.78	0.978
**Partial seizures**							
Baseline frequency	4	3.13 (6.80)		1	3.57 (9.45)		0.902
Day 1	4	1.93 (4.38)	38.34	1	4.57 (12.09)	−28.01	0.455
Day 2	4	1.00 (1.89)	68.05	1	3.86 (10.21)	−8.12	0.295
Day 3	2	0.27 (0.70)	91.37	1	4.00 (10.58)	−12.04	0.177
Day 4	0	0.00 (0.00)	100.00	1	3.86 (10.21)	−8.12	0.147
Day 5	0	0.00 (0.00)	100.00	1	3.43 (9.07)	3.92	0.147
Week 1	0	0.00 (0.00)	100.00	1	4.00 (10.58)	−12.04	0.147
Week 2	4	0.73 (1.49)	76.68	1	3.14 (8.32)	12.04	0.278
Week 3	4	0.93 (1.83)	70.29	1	3.00 (7.94)	15.97	0.339
Week 4	4	2.00 (3.55)	36.10	1	4.00 (10.58)	−12.04	0.510

*p < 0.05, **p < 0.01, ***p < 0.001.

### Epileptiform Discharges

The repeated-measures ANOVA using the epileptiform discharges as the dependent variable revealed significant main effects for group [*F* (1, 20) = 115.657; *p* < 0.001], time [*F* (5, 20) = 18.414; *p* < 0.001], and group × time interaction [*F* (5, 20) = 19.078; *p* < 0.001].

**Figure 3:**
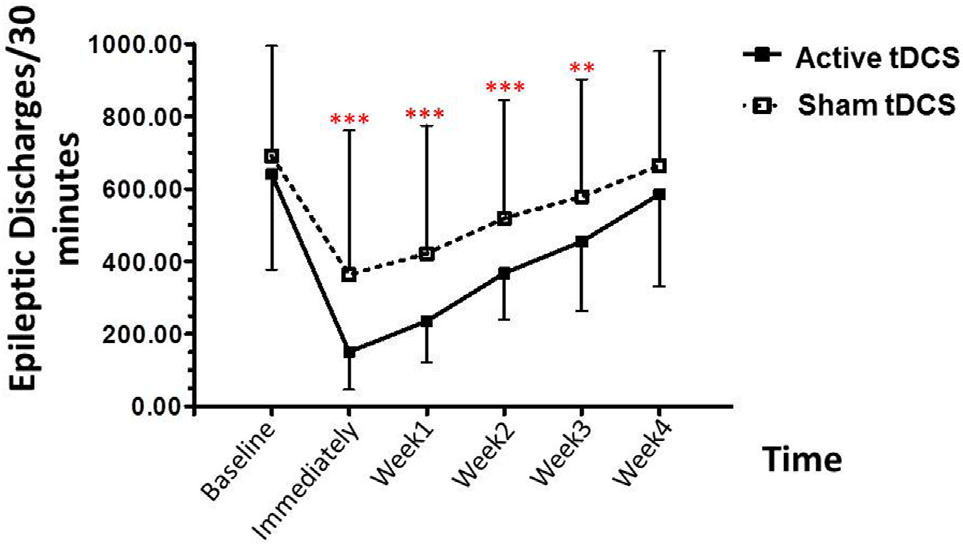
**Effect of tDCS on epileptiform discharges**. Data are presented as mean ± SD of number epileptiform discharges per 30 min of EEG recording at baseline, and various time points after treatment: 1, 2, 3, and 4 weeks. **p = 0.005 and ***p < 0.001 as compared to sham tDCS condition.

*Post hoc* analysis showed that the epileptiform discharges were significantly decreased in the tDCS group, relative to the sham tDCS group during the treatment period, at immediately post treatment (*p* < 0.001), 1 week (*p* < 0.001), 2 weeks (*p* < 0.001), 3 weeks (*p* = 0.005), and 4 weeks (*p* = 0.090) (Figure 3).

The epileptiform discharges (EDs) counted in our study consist of all spikes, sharp waves, spike waves, and slow waves. Visual EEG analysis, in the active tDCS group, the baseline for EDs was 640.13 ± 263.30 events/30 min, decreased to 150.53 ± 104.80 events/30 min (76.48% reduction) immediately after stimulation. After the treatment, the EDs were 235.73 ± 114.01 events/30 min (63.17% reduction), 367.07 ± 127.02 events/30 min (42.66% reduction), and 585.33 ± 127.02 events/30 min (8.56% reduction) in 1-, 2-, 3-, and 4-week follow-up, respectively.

### Vital Signs and Oxygen Saturation

All of the patients were no clinically relevant difference between vital signs and oxygen saturation during the treatment and 30 min after the treatment period.

## Discussion

This is the first RCT examining the efficacy of cathodal tDCS when combined with standard care in the treatment of patients with LGS. Consistent with the study hypotheses, the primary outcome revealed a significantly greater pre- to post-treatment decrease in seizure frequency that maintained for 4 weeks among participants in the active tDCS, relative to those in the sham tDCS condition. However, the statistical reductions in mean numbers of seizures were found in only tonic, atonic, and absence seizures. We also found statistically significant between-group differences in the secondary outcome variables emerged for epileptic discharges for 3 weeks post-treatment. In addition, we also found significantly clinical seizure frequency reduction after the treatment and extended to 4 weeks.

To the best of our knowledge, this is the first study evaluating cathodal tDCS in patients with LGS, a comparison with previous results of tDCS in this patient population is not possible. However, previous studies have shown the beneficial effects of cathodal tDCS in patients with other epilepsy conditions, especially the antiepileptic effect occurred immediately after stimulation (17, 18, 24, 25). In addition, our results are consistent with our previous study, which revealed a significant epileptiform discharges reduction following a single cathodal tDCS over the epileptic focus in children with refractory focal epilepsy (18). However, the duration of the antiepileptic effect in this study was longer, e.g., epileptic discharge waves were suppressed for up to 3 weeks, while our previous study revealed a significant epileptiform discharges reduction for 48 h after treatment. Possible explanations for this discrepancy are the longer duration (e.g., five consecutive days vs. single dose) and the higher current density (e.g., 2 vs. 1 mA) of treatment by cathodal tDCS could be more potential effect on epileptic neurons.

The mechanisms of cathodal tDCS on anti-epileptic effect have not been clearly understood. However, the basic knowledge of seizure generation causes from three hallmarks, hyper-excitability of neuron disinhibition and hypersynchrony of neural circuits (15, 26). Since cathodal tDCS induces a weak-constant electrical current, which increases resting membrane potential of neuronal cells. This action leads to decrease overall firing activity in the cortical areas immediately below the cathodal electrode (26). Therefore, it is possible that cathodal tDCS may inhibit the hyperexcitability of epileptic neurons. According to the long lasting effect of cathodal tDCS on the brain thought to be mediated by *N*-methyl-d-aspartate (NMDA) receptors with a long-term depression (LTD) fashion at the synaptic level (15). As well as the alteration in trans-membrane protein and changes in membrane pH could be also possible to induce LTD (27).

An important limitation of the current study is the standard procedures for electrode placement, although we used the international 10–20 system for tDCS electrode placement but we could not ensure that the stimulation electrode was directly over the M1. To specify the M1 site by, i.e., using transcranial magnetic stimulation (TMS) to locate motor responses is suggested (28, 29). However, it could increase risk of the seizure attack in this vulnerable population. In addition, the sample size of this study was small; therefore, it could have incurred a type II error when reporting the results for the seizure frequency. Besides, the population of this study is not homogeneous regarding the etiology; it might also be able to bias the results.

In summary, to the best of our knowledge, this is the first study to demonstrate that cathodal tDCS over the motor cortex when combined with standard treatment have beneficial anti-epileptic effect for at least 3 weeks. Further research is needed to examine these effects in the potential mechanisms of treatment using neuroimaging techniques.

## Author Contributions

NA: treatment, EEG analysis. KS: case collection. OT: statistic analysis. PA: study design, tDCS.

## Conflict of Interest Statement

The authors declare no financial or personal conflicts of interest associated with this study.
