# Supplementary figures and images for: Transcranial Direct Current Stimulation for Treatment of Childhood Pharmacoresistant Lennox–Gastaut Syndrome: A Pilot Study
Source: Front Neurol. 2016 May 4;7:66. doi: 10.3389/fneur.2016.00066 (PMC4854865; doi:10.3389/fneur.2016.00066)

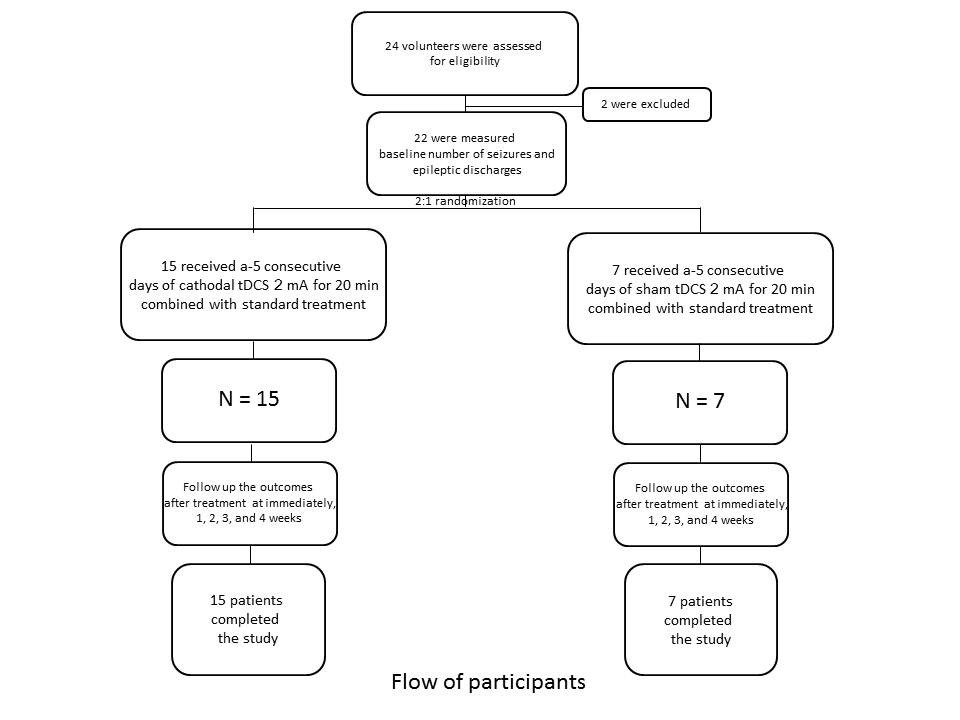

Supplement: Supplementary file 1 [file Image_1.JPEG]
